# No gene-by-environment interaction of BDNF *Val66Met* polymorphism and childhood maltreatment on anxiety sensitivity in a mixed race adolescent sample

**DOI:** 10.1080/20008198.2018.1472987

**Published:** 2018-05-22

**Authors:** Lindi Martin, Sian Megan Joanna Hemmings, Martin Kidd, Soraya Seedat

**Affiliations:** a Department of Psychiatry, Stellenbosch University, Cape Town, South Africa; b Department of Statistics and Actuarial Science, Stellenbosch University, Cape Town, South Africa

**Keywords:** Anxiety sensitivity, childhood trauma, childhood maltreatment, adolescents, genetics, gene-environment, BDNF *Val66Met*, sensibilidad ansiosa, trauma infantil, maltrato infantil, adolescentes, genética, gen-ambiente, Val66Met del BDNF, 焦虑敏感度, 童年创伤, 童年虐待, 青少年, 遗传学, 基因 - 环境, BDNF Val66Met, • We assessed the interactive effect of the BDNF Val66Met polymorphism and childhood maltreatment on anxiety sensitivity in adolescents. • A main effect of childhood maltreatment on anxiety sensitivity was evident, however, no main effect of BDNF genotype on anxiety sensitivity was revealed. • No significant G x E (BDNF Val66Met by childhood maltreatment) effect on anxiety sensitivity was evident.

## Abstract

**Background**: Anxiety disorders in youth are attributable to multiple causal mechanisms, comprising biological vulnerabilities, such as genetics and temperament, and unfavourable environmental influences, such as childhood maltreatment (CM).

**Objective**: A gene-environment (G x E) interaction study was conducted to determine the interactive effect of the BDNF *Val66Met* polymorphism and CM to increase susceptibility to anxiety sensitivity (AS) in a sample of mixed race adolescents.

**Method**: Participants (*n =* 308, mean age = 15.8 years) who were all secondary school students and who completed measures for AS and CM were genotyped for the BDNF *Val66Met* polymorphism. Hierarchical multiple regression analysis was conducted to assess G x E influences on AS. Age and gender were included in the models as covariates as age was significantly associated with AS total score (*p <* .05), and females had significantly higher AS scores than males (*p <* .05).

**Results**: A main effect of CM on AS was evident (*p <* .05), however, no main effect of BDNF genotype on AS was observed (*p >* .05). A non-significant G x E effect on AS was revealed (*p <* .05).

**Conclusions**: Our results suggest that CM does not have a moderating role in the relationship between the BDNF *Val66Met* genotype and the increased risk of anxiety-related phenotypes, such as AS. Given the exploratory nature of this study, findings require replication in larger samples and adjustment for population stratification to further explore the role of BDNF *Val66Met* and CM on AS in mixed race adolescents.

## Abstract (es)

**Antecedentes:** los trastornos de ansiedad en los jóvenes son atribuibles a múltiples mecanismos causales, que comprenden vulnerabilidades biológicas, como la genética y el temperamento; y las influencias ambientales desfavorables, como el maltrato infantil (MI). **Objetivo:** Se realizó un estudio interacción gen-ambiente (GxA) para determinar el efecto interactivo del polimorfismo Val66Met del BDNF y MI para aumentar la susceptibilidad para la sensibilidad a la ansiedad (SA) en una muestra de adolescentes de raza mixta.

**Método:** los participantes (n=308, Edad Media:15.8 años) que fueron todos estudiantes de secundaria que completaron las medidas para SA y MI, fueron genotipificados para el polimorfismo Val66Met del BDNF. Se realizó un análisis de regresión múltiple jerárquica para evaluar las influencias GxA en SA. La edad y género se incluyeron como covariables en los modelos, ya que la edad se asoció significativamente con el puntuación total SA (p<0.05), y las mujeres tuvieron puntuaciones de SA significativamente mayores que los hombres (p<0.05).

**Resultados:** Un efecto principal de MI en SA fue evidente (p<0.05), sin embargo, no se observó ningún efecto principal del genotipo BDNF en SA (p>0.05). Se reveló un efecto GxA No significativo sobre SA (p<0.05).

**Conclusiones:** Nuestros resultados sugieren que MI no tiene un rol moderador en la relación entre el genotipo Val66Met del BDNF y el mayor riesgo de fenotipos relacionados con la ansiedad, como SA. Dada la naturaleza exploratoria de este estudio, los hallazgos requieren la replicación en muestras más grandes y el ajuste de la estratificación de la población para explorar más a fondo el rol de Val66Met del BDNF y MI en SA en adolescentes de raza mixta.

## Abstract (zh)

**背景：**青年时期的焦虑症可归因于多种因果机制，包括生物学上的易感性（如遗传和气质）; 和不利的环境影响，如童年虐待（CM）。

**目的：**分析基因-环境（G×E）相互作用研究以确定BDNF Val66Met多态性与CM在混合种族青少年样本中增加对焦虑敏感性（AS）的易感性的交互作用。

**方法：**被试（*n =* 308，平均年龄：15.8岁）都是中学生，并完成了AS和CM测量，进行了BDNF Val66Met多态性的基因型分类。进行层级多元回归分析以评估G×E对AS的影响。 年龄和性别作为协变量包括在模型中，因为年龄与AS总分显著相关（p <0.05），女性AS评分显著高于男性（p <0.05）。

**结果：**CM对AS有明显主效应（p <0.05），但BDNF基因型对AS的主效应没有观察到（p> 0.05）， 说明了GxE对 AS的影响不显著（p <0.05）。

**结论**：我们的研究结果表明，CM没有调节BDNF Val66Met基因型与焦虑相关表型（如AS）风险增加之间的关系。 鉴于本研究的探索性质，研究结果需要在更大的样本中重复，并调整人群分层，以进一步探索在青少年混合种族样本中BDNF Val66Met和CM对AS的作用。

## 1. Background

Anxiety disorders in youth are attributable to multiple causal mechanisms, comprising biological vulnerabilities, such as genetics and temperament, and unfavourable environmental influences (Broekman, 2011; Dabkowska & Dabkowska-Mika; Keeley & Storch, 2009; Weems & Stickle, 2005), such as childhood maltreatment (CM) and trauma (Al-Hadethe, Hunt, Thomas, & Al-Qaysi, 2014; Dvir, Ford, Hill, & Frazier, 2014; Nordanger et al., 2014). Temperament or dispositional characteristics may be considered intermediate phenotypes for psychiatric disorders, reflecting sub-threshold clinical presentations (Altınbaş et al., 2015). Anxiety-related temperamental traits, such as anxiety sensitivity (AS) and trait anxiety (Eysenck, 1992; Reiss, Peterson, Gursky, & McNally, 1986), have consistently been found to be predictive of anxiety disorders and symptoms in youth (McLaughlin, Stewart, & Taylor, 2007; Muris, Schmidt, Merckelbach, & Schouten, 2001; Schmidt, Zvolensky, & Maner, 2006; Schmidt et al., 2010; Weems et al., 2007), particularly panic disorder symptoms (Hayward, Killen, Kraemer, & Taylor, 2000; Schmidt et al., 2006). Such temperamental traits are considered developmentally stable risk factors for anxiety (Garcia et al., 2013; Zavos, Gregory, & Eley, 2012; Zavos, Rijsdijk, & Eley, 2012).

AS refers to fear of anxiety-related or arousal-related sensations and symptoms (Reiss & McNally, 1985) due to erroneous or dysfunctional beliefs about the consequences of such symptoms (Reiss et al., 1986). Individuals with elevated levels of anxiety-related temperamental traits, such as those with high levels of AS, may be termed ‘anxiety prone’, relative to those individuals with normative levels of anxiety (Simmons, Strigo, Matthews, Paulus, & Stein, 2006).

AS is moderately heritable, in the range 34–45% in youth and adults (Eley, Gregory, Clark, & Ehlers, 2007; Stein, Jang, & Livesley, 1999; Zavos, Rijsdijk, Gregory, & Eley, 2010). Moreover, AS is thought to be interactively impacted by additive genetic factors as well as unique environmental factors (Garcia et al., 2013; Stein et al., 1999), such as stressful life events (Aktekin et al., 2001; Zavos et al., 2012), including CM and severe family conflict (McLaughlin & Hatzenbuehler, 2009; Scher & Stein, 2003), demonstrating the interactive effect of genes and environment on AS.

Brain-derived neurotrophic factor (BDNF) is a secretory protein in the neurotrophin family known to influence the proliferation, survival, differentiation, repair and regulation of synaptic plasticity of neuronal cells in the developing and adult brain (Bath & Lee, 2006; Chen et al., 2006; Martinowich, Manji, & Lu, 2007; Notaras, Hill, & van den Buuse, 2015). BDNF is widely expressed in the hippocampus and cerebral cortex (Hofer, Pagliusi, Hohn, Leibrock, & Barde, 1990; Huang & Reichardt, 2001) and enhances hippocampal long-term potentiation (Figurov, Pozzo-Miller, Olafsson, Wang, & Lu, 1996) associated with both memory and learning efficiency (Hariri et al., 2003; Yamada, Mizuno, & Nabeshima, 2002). The gene encoding BDNF contains a functional single-nucleotide polymorphism (SNP) resulting in a valine (val) to methionine (met) substitution at amino acid 66 (*Val66Met*, rs6265) in the 5ʹ pro-BDNF domain (Egan et al., 2003). Compared with the *Val66* allele, the *Met66* allele is associated with a decrease in activity-dependent secretion of BDNF (Egan et al., 2003). BDNF has received attention due to its evident role in anxiety and mood disorders (Angelucci et al., 2014; Hemmings et al., 2008; Li, Chang, & Xiao, 2016; Martinowich et al., 2007; Molendijk et al., 2014; Suliman, Hemmings, & Seedat, 2013), although findings have been inconsistent across studies (Frustaci, Pozzi, Gianfagna, Manzoli, & Boccia, 2008; Hong, Liou, & Tsai, 2012; Lam, Cheng, Hong, & Tsai, 2004; Minelli et al., 2011; Notaras et al., 2015; Surtees et al., 2007; Wang et al., 2015).

Some studies have reported associations between the BDNF *Val66Met* polymorphism and personality traits such as trait anxiety and neuroticism (Lang et al., 2005; Sen et al., 2003), while others have not (Terracciano et al., 2010; Willis-Owen et al., 2005). Previous studies have found associations between the *Met66* allele and increased introversion (Terracciano et al., 2010), harm avoidance (Montag, Basten, Stelzel, Fiebach, & Reuter, 2010), tendency to ruminate (Beevers, Wells, & McGeary, 2009), lower levels of conscientiousness (Hiio et al., 2011), an increased vulnerability to stress (Casey et al., 2009), increased anxiety-related behaviours (Chen et al., 2006) and increased risk of an anxiety disorder in children and adolescents (Tocchetto et al., 2011). In contrast, other studies have reported an association between the *Val66* allele and higher neuroticism scores, as well as increased risk of anxiety symptoms during adolescence (Chen, Yu, Liu, Zhang, & Zhang, 2015; Frustaci et al., 2008).

Studies have demonstrated that acute and chronic stress (e.g. military training, psychological job stress, acute and repeated restraint in animals) are associated with decreased BDNF (Mitoma et al., 2008; Murakami, Imbe, Morikawa, Kubo, & Senba, 2005; Suzuki et al., 2014) and enhancement of anxiety-related behaviours (Chen et al., 2006). In adults with lifetime major depressive disorder, for example, a linear relationship between exposure to CM (i.e. emotional neglect, psychological abuse, sexual abuse) and reduced BDNF serum levels has been demonstrated in *Met66* allele carriers, with the lowest BDNF levels evident in *Met66* carriers reporting two or more childhood trauma types (Elzinga et al., 2011). Similarly, compared with non-trauma exposed children and adolescents, youth with CM histories (i.e. sexual abuse) have been found to exhibit significantly lower serum BDNF levels, with the lowest levels documented in those with multiple sexual assault histories (Şimşek, Yüksel, Kaplan, Uysal, & Alaca, 2015).

There is support for the role of gene-environment interaction in the aetiology of youth and adult anxiety, including panic and separation anxiety, anxiety symptoms, anxious mood and anxious temperament (Baumann et al., 2013; Chen et al., 2015; Gunthert et al., 2007; Ibarra et al., 2014; Klauke et al., 2011; Lau, Gregory, Goldwin, Pine, & Eley, 2007; Stein, Schork, & Gelernter, 2008; Vendlinski, Lemery-Chalfant, Essex, & Goldsmith, 2011). CM and AS have both consistently been implicated as risk factors for psychopathology, including anxiety disorders (Collishaw et al., 2007; Kessler, McLaughlin et al., 2010; McLaughlin et al., 2007, 2012). We have previously determined, in a sample of secondary school-attending adolescents, that CM is significantly and positively associated with anxiety-related temperamental traits such as AS and trait anxiety (Martin, Viljoen, Kidd, & Seedat, 2014). Further, there is evidence that interactions of life stress/early adversity (including abuse and neglect) with BDNF *Val66Met* gene variants predict subsequent risk for psychopathology, such as depression, in youth and adults (Carver, Johnson, Joormann, Lemoult, & Cuccaro, 2011; Chen, Li, & McGue, 2013; Gutiérrez et al., 2015; Hosang, Shiles, Tansey, McGuffin, & Uher, 2014). G x E interactions (i.e. BDNF *Val66Met* and early life stress exposure) have previously been found to be associated with abnormalities in brain structures, physiological indicators, deficits in cognition (i.e. poorer working memory), higher levels of depression and anxiety, and elevated temperamental traits (Gatt et al., 2009). However, data on the interactive effects of CM and genetic factors, such as the BDNF *Val66Met* polymorphism, on susceptibility to anxiety-related temperamental traits, such as AS, in adolescents is limited. Identifying risk markers for AS, an intermediate phenotype for anxiety disorders, in non-clinical samples is crucial to informing community- and school-level prevention strategies. Early and accurate identification is even more important in light of the wide mental health treatment gap that exists in many country settings. To our knowledge, no previous studies have investigated whether the BDNF *Val66Met* polymorphism interacts with CM to increase susceptibility to AS in adolescents. In addition, relative to studies that have assessed the BDNF *Val66Met* polymorphism in Caucasian samples, there is limited information of the allelic distribution of the BDNF *Val66Met* polymorphism in ethnically diverse samples. A G x E interaction study was conducted to determine the interactive effect of the BDNF *Val66Met* polymorphism and CM to increase susceptibility to AS in a school sample of South African mixed race adolescents.

## 2. Method

### 2.1. Design

This study was a two-tier study in a sample of secondary school students. Tier 1 employed stratified two-stage cluster sampling in which public secondary schools in Cape Town, South Africa, and students within these schools, were randomly selected. The Tier 1 sample of secondary school students was therefore representative of students attending public secondary schools in Cape Town, South Africa. Tier 1 allowed for the screening (e.g. of levels of CM and AS) and collection of salivary DNA from secondary school students from 29 public schools in Cape Town, South Africa. A description of Tier 1 methods, including clinical data pertaining to Tier 1, has previously been reported (Martin et al., 2014). Four groups of adolescents grouped according to levels of Tier 1 self-reported CM and AS, amongst others, and matched as closely as possible on age, ethnicity, gender and educational status, were included in the second tier of the study and underwent a neuropsychological and neuroimaging assessment, amongst others.

### 2.2. Participants

The Tier 1 sample consisted of 1149 secondary school students. DNA was extracted from 985 participants (i.e. comprising 85.7% of the total Tier 1 sample) at the same time that Tier 1 self-report measures (e.g. CM and AS) were administered. The majority of the Tier 1 sample consisted of Black (64.4%) and mixed race (32.2%) participants. As almost all Black participants (99.68%, 628/630) in the sample were *Val66Val* genotype carriers, the subset reported on here included only those participants that self-classified as ‘mixed race’ and for whom BDNF data were available (*n =* 308).

This study was approved by the Health Research Ethics Committee of Stellenbosch University, South Africa (ethics reference number: N10/11/370), and permission to access secondary schools and conduct this study was provided by the Western Cape Education Department. Written informed consent was obtained from parents/legal guardians and written assent was obtained from the students themselves.

### 2.3. Self-report measures

The following self-report questionnaires, amongst others, were administered at the secondary schools on a single occasion.

The Childhood Anxiety Sensitivity Index (CASI; Silverman, Fleisig, Rabian, & Peterson, 1991) is an 18-item instrument that measures the fear of anxiety symptoms, designed for use with school-age children and adolescents. The CASI yields a total score that can range from 18 to 54, with higher scores reflecting higher levels of AS.

The Childhood Trauma Questionnaire – Short Form (CTQ-SF; Bernstein et al., 2003) is a brief, 28-item, retrospective self-report measure of the frequency of abuse (i.e. emotional, physical, sexual) and neglect (i.e. emotional, physical) experienced prior to age 18 years. The CTQ-SF yields a total score in the range 25–125, with higher scores reflecting more severe levels of abuse or neglect. Scores for each of the CTQ-SF subscales are in the range 5–25, with higher scores indicating more severe childhood trauma (Bernstein & Fink, 1998). For the purpose of the current study, we enquired about abuse and neglect experienced prior to age 12 years.

### 2.4. Genotyping

Genomic DNA was extracted from saliva collected in Oragene DNA self-collection kits (OG-500, DNA Genotek, Ontario, Canada) using the Prep-It L2P reagent (DNA Genotek) as per manufacturer’s instructions. The BDNF *Val66Met* polymorphism (rs6265) was genotyped as previously described (Hemmings et al., 2008).

### 2.5. Data analysis

All analyses were conducted using STATISTICA version 13 (StatSoftInc., 2015). Univariate normality was determined for all demographic and clinical variables. Demographic characteristics of the total sample (i.e. age, gender, current grade at school), as well as self-report variables of interest (i.e. AS, CTQ), were calculated as means (*M*) and standard deviations (*SD*) for quantitative variables and counts and associated percentages for categorical variables (e.g. gender). The severity (i.e. minimal, moderate, severe, extreme) of abuse and neglect categories of the CTQ (i.e. emotional, physical and sexual abuse, emotional and physical neglect) were computed for the total sample using recommended cut-off scores (Bernstein & Fink, 1998).

To determine if AS levels differed by gender and age, *t*-tests and Pearson’s correlation statistic, respectively, were employed. Genotype counts (%) and the Hardy-Weinberg equilibrium (HWE) *p*-value was determined using the R Package SNPassoc (González et al., 2007). Demographic and clinical characteristics of the sample by genotype (i.e. *Val66* homozygotes and *Met66* allele carriers) were summarized as means (*M*) and standard deviations (*SD*) for quantitative variables, and counts were used for categorical variables. Independent samples *t*-tests and chi-square tests were used to determine any group (i.e. genotype) differences for the quantitative and categorical variables, respectively. Pearson’s correlation statistic was used to assess the relationship between CTQ total score and CASI total score by genotype.

Hierarchical multiple regression analysis was used to assess the effects of BDNF genotype (coded 0 for *Val66Val* and 1 for *Met66* allele carriers), level of CM and the interaction of BDNF genotype and CM, on AS. In the first model, gender, age, BDNF genotype and CTQ total score were included to assess the main effects of BDNF genotype and CTQ total score on the outcome, AS. In the second model, the two-way interaction term (i.e. BDNF x CTQ) was added. The *F*-to-remove test was used to compare the *R^2^* change between the first and second model to determine whether the inclusion of the interaction term resulted in a significant increase in explained variance.

## 3. Results

### 3.1. Demographic and clinical variables

All participants were secondary school students (mean: grade 10; range: grades 8–10). The mean age of the sample was 15.8 years (*SD *= 1.59; range: 12–20 years). Over half the sample was female (183/308, 59.4%).

The mean CASI score for the total sample was 34.10 (*SD *= 6.81, range: 18–53). The mean CTQ score for the total sample was 43.73 (*SD* = 14.54, range: 25–96). Mean scores for the CTQ subscales for the total sample were as follows: emotional abuse: *M *= 10.54 (*SD *= 4.78), physical abuse: *M *= 7.58 (*SD *= 4.01), sexual abuse: *M *= 7.04 (*SD *= 4.09), emotional neglect: *M *= 10.61 (*SD *= 4.46), physical neglect: *M *= 7.96 (*SD *= 3.34) (see Table 1 for frequencies and percentages of abuse and neglect types endorsed by the sample). Emotional abuse was the most frequently reported CM type (i.e. 57.4% of the sample reportedly experienced low to extreme forms of emotional abuse), followed by emotional neglect (i.e. 49.9% of the sample reportedly experienced low to extreme forms) and physical neglect (i.e. 43.8% low to extreme forms). Age was significantly associated with AS (*r *= 0.17, *p <* .05), and females had significantly higher AS scores than males [males: *M *= 31.58, *SD *= 6.26; females: *M *= 35.83, *SD *= 6.64, (*t*(306) = −5.63, *p <* .05)].

**Table 1.** Frequencies and percentages of abuse and neglect categories in the total sample (*n =* 308).

Childhood trauma category	None or minimal	Low to moderate	Moderate to severe	Severe to extreme
Emotional abuse	131 (42.5%)	87 (28.2%)	44 (14.3%)	46 (14.9%)
Physical abuse	211 (68.5%)	42 (13.6%)	23 (7.5%)	32 (10.4%)
Sexual abuse	202 (65.6%)	36 (11.7%)	39 (12.7%)	31 (10.1%)
Emotional neglect	154 (50%)	90 (29.2%)	38 (12.3%)	26 (8.4%)
Physical neglect	173 (56.2%)	61 (19.8%)	38 (12.3%)	36 (11.7%)

### 3.2. Genetic variables

The BDNF *Val66Met* SNP was in Hardy-Weinberg equilibrium (*p *= .456). The following genotype frequencies were evident in our mixed race sample: *Val66Val* (75.65%, 233/308), *Val66Met* (22.08%, 68/308) and *Met66Met* (2.3%, 7/308). These frequencies are generally in line with those determined in Caucasian samples (Carver et al., 2011; Gatt et al., 2009; Pivac et al., 2009; Surtees et al., 2007; Zeni et al., 2013) and in South African mixed race samples (Dalvie et al., 2014), and confirm the low rates of *Met66* allele carriers evident in ethnic groups in sub-Saharan Africa (Petryshen et al., 2009). Given the low frequency of *Met66Met* genotype carriers, *Val66Met* and *Met66Met* genotypes were combined (24.35%, 75/308) for genotypic analyses to increase statistical power. No significant differences in either demographic [i.e. age and grade at school (*p >* .05)] or self-report measures [i.e. CASI and CTQ (*p >* .05)], by genotype, were evident. No association between gender and genotype was evident [*Χ*^2^ (1, *N =* 308) = 0.480, *p >* .05]. The relationship between CTQ and CASI was relatively stronger in *Met66* allele carriers (*r *= 0.48, *p <* .01) than in *Val66* homozygotes (*r *= 0.32, *p <* .01) (see Table 2 for demographic and self-report variables by genotype).

**Table 2.** Summary statistics for demographic and clinical variables by BDNF genotype.

	Genotype		
	*Val66Val* (*N =* 233)	*Met66Met* + *Val66Met* (*N =* 75)	
Variable	Mean (*SD*)	Mean (*SD*)	*p*-value
Age	15.83 (1.56)	15.79 (1.71)	> .05
Grade	9.86 (1.31)	9.98 (1.36)	> .05
CASI	34.22 (6.75)	33.73 (7.01)	> .05
CTQ	44.18 (14.78)	42.36 (13.78)	> .05

CASI = Childhood Anxiety Sensitivity Index; CTQ = Childhood Trauma Questionnaire

Results of multiple regression analyses (i.e. model 1 and model 2) are presented in Table 3. A significant main effect of CTQ total score (*p <* .01) on AS was evident, however, no significant main effect of BDNF genotype on AS was observed (*p >* .05). In the second model, the inclusion of the interaction term did not contribute to an increase in explained variance (*p >* .05).

**Table 3.** Regression analysis depicting main and interaction effects.

Model	Predictors	ß	*t* (*p*)	*R*	*R^2^*	Δ*R^2^*	Δ*F* (*p*)
1	Gender	0.29	5.83*	0.488	0.239		
	Age	0.15	2.93*				
	BDNF *Met66*	0.00	0.01				
	CTQ total	0.34	6.82*				
2	Gender	0.30	5.76*	0.493	0.243	0.004	1.739 (> .05)
	Age	0.15	2.91*				
	BDNF *Met66*	−0.20	−1.25				
	CTQ total	0.31	5.44*				
	BDNF *Met66* x CTQ total	0.21	1.32				

BDNF *Met66* = BDNF *Met66* allele carriers vs. BDNF *Val66* homozygotes; ß = standardized regression coefficient;

*t* (*p*) = *t*-statistic and associated *p*-value; *p < .05; *R* = correlation statistic; *R^2^* = explained variance.

## 4. Discussion

Anxiety-related temperamental traits, such as AS, trait anxiety and neuroticism, are said to be interactively impacted by genetic and environmental factors, such as CM and early life stress and trauma. This study investigated whether the BDNF *Val66Met* polymorphism interacted with CM to increase susceptibility to AS in a sample of mixed race adolescents. To our knowledge, this is the first study to assess the role of BDNF *Val66Met* in AS in a South African mixed race sample of adolescents.

Our results revealed a significant main effect of CM on AS, however, there was no significant main effect of BDNF genotype on AS. These findings suggest that the BDNF *Val66Met* polymorphism does not have a direct effect on AS. This finding is in line with studies that have found no significant direct association between BDNF *Val66Met* polymorphism and personality traits such as neuroticism or harm avoidance and anxiety disorders or mood disorders, including OCD, panic disorder, PTSD and depression (Arias et al., 2012; Chen et al., 2013; Frustaci et al., 2008; Hong et al., 2012; Minelli et al., 2011; Surtees et al., 2007; Terracciano et al., 2010), despite some studies reporting such associations (Frustaci et al., 2008; Lang et al., 2005; Min et al., 2013; Montag et al., 2010; Sen et al., 2003; Terracciano et al., 2010). Firstly, the grouping of *Met66* allele carriers (i.e. *Met66Met* and *Val66Met* genotypes), as is frequently carried out in studies in which the rate of the *Met66Met* genotype is relatively low, such as in Caucasian samples (Gatt et al., 2009; Lehto, Maestu, Kiive, Veidebaum, & Harro, 2016; Nedic et al., 2013; Pivac et al., 2009), may introduce a bias in which a main effect of genotype is not detected due to the exclusion of the *Met66Met* genotype in analyses (Notaras et al., 2015). Secondly, the *Val66Met* and *Met66Met* genotypes, respectively, may have dissimilar effects (Hong et al., 2012). Nevertheless, the main effect of CM on AS determined in this study provides support for the positive association between CM, including stressful life events, and AS in adolescents (McLaughlin & Hatzenbuehler, 2009; Tollenaar, Molendijk, Penninx, Milaneschi, & Antypa, 2017), a well-established cognitive risk factor for the development of anxiety disorders and associated symptoms in youth (Hishinuma et al., 2001; McLaughlin et al., 2007; Muris et al., 2001). Furthermore, our finding adds to the well-established literature demonstrating the adverse acute and long-term effects of CM or trauma on mental health and cognition in youth and adults (De Bellis, Woolley, & Hooper, 2013; Greger, Myhre, Lydersen, & Jozefiak, 2015; Irigaray et al., 2013; Taillieu, Brownridge, Sareen, & Afifi, 2016; Teicher, Ohashi, Lowen, Polcari, & Fitzmaurice, 2015).

Our results revealed a non-significant BDNF genotype x CM effect on AS. This finding is not consistent with studies that found that the low-functioning BDNF *Met66* allele and CM/childhood trauma or early life adversity or stress interact to predict increased susceptibility for psychopathology, including anxiety-related temperamental traits, such as neuroticism (Gatt et al., 2009) and guilt-proneness (Szentágotai-Tətar et al., 2015), anxiety symptoms (Gatt et al., 2009) and mood disorders and associated symptoms (Aguilera et al., 2009; Carver et al., 2011; Gutiérrez et al., 2015). Our findings are also not in agreement with G x E studies that found an interactive effect of the higher functioning *Val66* allele and environmental exposures (i.e. adversity, negative stressors, recent life events) on psychopathology, such as increased levels of neuroticism, harm avoidance and depression (Chen et al., 2013; Kim et al., 2009; Lehto et al., 2016). Apart from the possible confounding effects of age and gender, discrepant results across studies may in part be due to population-driven differences in BDNF *Val66Met* frequencies, given that the *Met66* allele has consistently been found to be more common in Asian populations than in Caucasian populations (Chen et al., 2013; Petryshen et al., 2009). A further confounding factor may include phenotype heterogeneity and methodological (assessment) differences (Hong et al., 2012).

A number of study limitations should be taken into account in interpreting the current findings. First, due to the cross-sectional nature of this study, inferences about causality cannot be made. Second, our sample size of *N *= 308 is relatively small given the estimated sample sizes required in candidate gene studies in which functional polymorphisms are assessed and in which minor effects are expected (Duncan & Keller, 2011). Third, use of the CTQ, a retrospective self-report measure of CM, may have introduced recall bias which may have resulted in the over- or under-reporting of maltreatment frequency. Fourth, we explored one polymorphism within the BDNF gene. Finally, we did not correct for gene-environment correlation (rGEs) or population stratification. The South African mixed race population is characterized by high levels of admixture (Tishkoff et al., 2009) and ancestral diversity (i.e. Khoesan, European and Asian ethnicity) (Hemmings et al., 2016; Wright, Niehaus, Koen, Drögemöller, & Warnich, 2011), suggestive of genetic heterogeneity, which may have influenced our results. Findings of this exploratory study are therefore preliminary. Our findings add to the literature on aetiological processes that may underlie the development of anxiety-related traits, symptoms and disorder in adolescents. More specifically, our findings shed light on the role of the BDNF *Val66Met* polymorphism in the development of anxiety-related traits in mixed race adolescents in the context of childhood adversity. Furthermore, these results extend findings of the role of BDNF and CM on AS. Previous studies have focused on convenience samples of college students, or adults, and predominantly on the effects of the *5-HTTLPR* polymorphism (Hemmings et al., 2016; Kim et al., 2009; Klauke et al., 2011; Laucht et al., 2009; Stein et al., 2008). Our results suggest that the influence of CM on adolescent AS levels is not moderated by the BDNF *Val66Met* polymorphism. Our findings highlight the importance of assessing gene-environment interactions in the assessment of genetic effects on anxiety-related phenotypes associated with anxiety disorders. Recommendations for future research include replication in larger samples of mixed race participants in which population stratification is corrected for and in which the *Met66Met* genotype is better represented (Notaras et al., 2015). Furthermore, variation across the BDNF gene would be useful to consider (Mandelman & Grigorenko, 2012). Additionally, the effect of other genetic variants, in conjunction with the BDNF *Val66Met* polymorphism, should be explored given findings of an epistatic effect between BDNF and the serotonin transporter genes (Martinowich & Lu, 2008; Pezawas et al., 2008). Further support for the aforementioned is reflected in findings of gene x gene and gene x gene x environment interactions which are indicative of significant interaction effects of the BDNF *Val66Met* polymorphism and the serotonin transporter gene (*SLC6A4*) on anxiety-related traits (e.g. neuroticism and harm avoidance) (Arias et al., 2012; Terracciano et al., 2010) and depressive symptoms and disorder (Gutiérrez et al., 2015; Kaufman et al., 2006). Finally, the effects of environmental influences other than CM, such as parenting rearing practices (Ibarra et al., 2014) and general self-efficacy (Schiele et al., 2016), on AS should be explored. Investigation of the aforementioned would provide a clearer understanding of the genetic and environmental impacts on AS in mixed race adolescent samples.
